# Protocol for evaluation of enhanced models of primary care in the management of stroke and other chronic disease (PRECISE): A data linkage healthcare evaluation study.

**DOI:** 10.23889/ijpds.v4i1.1097

**Published:** 2019-08-05

**Authors:** NE Andrew, J Kim, DA Cadilhac, V Sundararajan, AG Thrift, L Churilov, NA Lannin, M Nelson, V Srikanth, MF Kilkenny

**Affiliations:** 1 Department of Medicine, Peninsula Clinical School, Central Clinical School, Monash University, Frankston, Victoria, Australia; 2 Stroke and Ageing Research, Department of Medicine, School of Clinical Sciences at Monash Health, Monash University, Clayton, Victoria, Australia; 3 Stroke Division, the Florey Institute of Neuroscience and Mental Health, The University of Melbourne, Heidelberg Victoria, Australia; 4 Department of Public Health, School of Psychology and Public Health, La Trobe University, Bundoora, Victoria, Australia; 5 School of Allied Health, Department of Community and Clinical Allied Health, La Trobe University, Melbourne, Victoria, Australia; 6 Menzies Institute for Medical Research, University of Tasmania, Hobart, Victoria, Australia

**Keywords:** Data Linkage, Primary Health Care, General Practitioner, Stroke, Chronic Disease, Secondary Prevention, Policy

## Abstract

**Introduction:**

The growing burden of chronic diseases means some governments have been providing financial incentives for multidisciplinary care and self-management support delivered within primary care. Currently, population-based evaluations of the effectiveness of these policies are lacking.

**Aim:**

To outline the methodological approach for our study that is designed to evaluate the effectiveness (including cost) of primary care policies for chronic diseases in Australia using stroke as a case study.

**Methods:**

Person-level linkages will be undertaken between registrants from the Australian Stroke Clinical Registry (AuSCR) and (i) Government-held Medicare Australia claims data, to identify receipt or not of chronic disease management and care coordination primary care items; (ii) state government-held hospital data, to define outcomes; and (iii) government-held pharmaceutical and aged care claims data, to define covariates. N=1500 randomly selected AuSCR registrants will be sent surveys to obtain patient experience information. In Australia, unique identifiers are unavailable. Therefore, personal-identifiers will be submitted to government data linkage units. Researchers will merge the de-identified datasets for analysis using a project identifier. An economic evaluation will also be undertaken.

**Analysis:**

The index event will be the first stroke recorded in the AuSCR. Multivariable competing risks Poisson regression for multiple events, adjusted by a propensity score, will be used to test for differences in the rates of hospital presentations and medication adherence for different care (policy) types. Our estimated sample size of 25,000 patients will provide 80% estimated power (ɑ>0.05) to detect a 6-8% difference in rates. The incremental costs per Quality-adjusted life years gained of community-based care following the acute event will be estimated from a health sector perspective.

**Conclusion:**

Completion of this study will provide a novel and comprehensive evaluation of the effectiveness and cost-effectiveness of Australian primary care policies. Its success will enable us to highlight the value of data-linkage for this type of research.

## Introduction

The global burden of stroke is large and increasing [1, 2]. In developed countries the contribution of Disability Adjusted Life Years (DALYs) due to stroke have been increasing proportionally to other diseases with concomitant burdens on healthcare systems [1] as stroke rarely occurs in isolation. Survivors have an increased risk of future cardiovascular events (approximately 11% at 1 year, 26% at 5 years) due to factors that precipitated the initial stroke event [3]. Stroke is also associated with considerable long-term costs from ongoing disability and associated comorbidity [4, 5]. Because of these complexities, models of care best suited to addressing patients’ ongoing care needs will vary. Some may only require secondary prevention management, whereas others may also require a range of other therapies and / or support. Regardless, all require some form of comprehensive long-term healthcare management.

Enhanced models of primary care, that included chronic disease management and care coordination, were introduced in Australia in 1999 to promote multidisciplinary care and self-management support within a continuous care model integrated across healthcare sectors. These additional Medicare claim items are funded by the federal government and so are available to people living in all states and territories. These care options may provide additional incentives to healthcare providers to ensure patients with stroke are managed according to recommended stroke clinical guidelines through comprehensive management of secondary prevention and disability [6]. However, long-term management and uptake of these care types appears to be sub optimal, with only 46% of patients aged over 65 years being put on these plans [7].

Evidence regarding the effectiveness of financial incentives such as these in primary care is conflicting [8]. Incentives have been shown to increase adherence to clinical indicators [9]. However, it is unclear if this is a consequence of improved recording required for financial gain [9]. The impact on patient outcomes is less clear. Preliminary evidence in Australia suggests that the use of chronic disease management primary care items can improve adherence to recommended care for diabetes [10] and reduce hospital readmissions for heart failure in Veteran populations [11]. However, a large scale comprehensive evaluation of the effectiveness of these primary care models has not been performed. In this paper we describe the protocol of the study ‘Evaluation of Enhanced Models of Primary Care in the Management of Stroke and Other Chronic Disease’ (PRECISE), a large scale data linkage project designed to address this knowledge gap. The study is funded by the National Health and Medical Research Council of Australia (GNT 1141848).

The main aims of the proposed study are to:

1. Compare differences in long-term health outcomes (survival, hospital contacts, quality of life) for survivors of stroke, with and without disability, based on the primary care based chronic disease management and / or care coordination they did or did not receive;
2. Compare differences in adherence and persistence of secondary prevention medications between patients with stroke who did and did not receive chronic disease management plans and / or care coordination; and
3. Determine the cost-effectiveness of different models of primary care received by those with and without disability over a two year period from 6 months following stroke onset.

The objectives of this paper are to describe:

1. Our methodological approach for use in other similar observational healthcare evaluation studies;
2. The scope and breadth of datasets that will be linked to highlight the utility of such datasets for evaluation purposes; and
3. Our proposed statistical and economic analysis plan.

## Methods

### Study design

**Figure 1: Study design and study observation periods.**
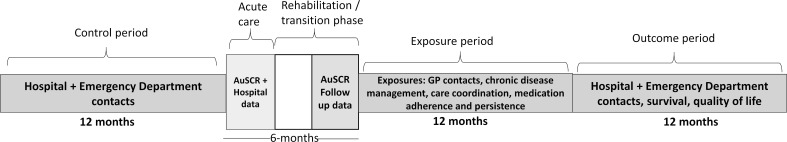
AuSCR: Australian Stroke Clinical Registry; GP: General Practitioner

The study is a comparative effectiveness and cost-effectiveness study using an observational, cohort-based design (Figure 1). Study outcomes will be reported according to the REporting of studies Conducted using Observational Routinely-collected health Data (RECORD) statement [12].

### Population

The cohort will be derived from the Australian Stroke Clinical Registry (AuSCR) and will include all registrants aged 18 years and over who were admitted to participating hospitals in Victoria and Queensland (n=42 hospitals) between 2012 and 2016 (approximately 25,000 registrants). This will allow for generalisability of the results across rural, regional and metropolitan settings and system level comparisons between two states whereby a large proportion (i.e. 45%) of the Australian population live. Those living in residential care (or aged care) will be excluded from the study as they are ineligible to receive the chronic disease management and care coordination primary care items that we are evaluating.

### Intervention

The intervention being evaluated is Australian Medicare funded enhanced primary care items. General Practitioners (GPs), also known as primary care physicians, are able to claim these Medicare items if they wish to provide the types of care outlined in Table 1. These invoke additional payments compared to a standard consultation to incentivise more comprehensive care and to compensate GPs for the additional time involved. The items available are: (1) initiation of a chronic disease management plan (item 721); (2) initiation or involvement in coordinated care (items 723 and 729); (3) review of a chronic disease or coordinated care plan (item 732); (4) initiation of a mental health management plan (items 2700, 2701, 2715 and 2717); and (5) review of a mental health management plan (item 2712). See Table 1 for a detailed description of these items.

**Figure 2: General practitioner pathways.**
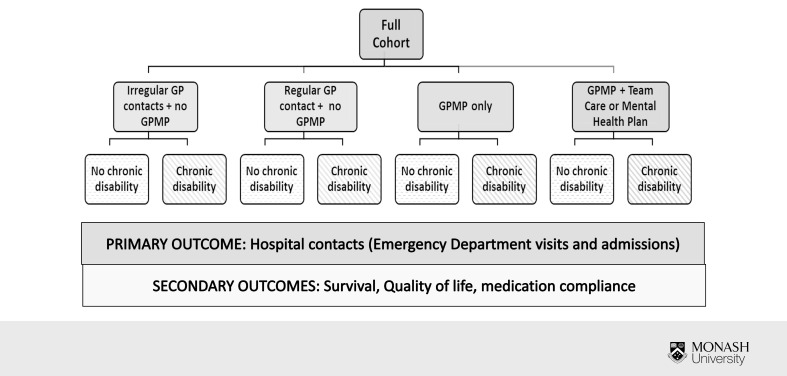
GP: General Practitioner

In addition, a Team Care Arrangement can be used to enable access to allied health professions at non-government funded clinics with costs associated with the first five visits in a calendar year heavily subsidised by Medicare [14]. Although some patients will access allied health services through other pathways, including private health insurance, a Team Care Arrangement should still be established to facilitate coordinated care. If a patient specifically requires care coordination for mental health problems, a GP Mental Health Care Plan (Table 1) can be used instead of a Team Care Arrangement. A chronic disease plan must be established prior to initiating a coordinated care plan. We have outlined the potential care pathways in Figure 2.

**Table 1: Chronic disease management and care coordination Medicare Australia items [13]:** GP, General Practitioner; * Item number refers to the Medicare item code.

Item name	Item number	Item description
Items used in defining use of Chronic Disease Management items (Exposure Cohort 1)		

Preparation of a Chronic Disease Management Plan	721	Must involve a comprehensive written plan describing: Healthcare needs; Management goals developed with the patient; Actions planning and strategies to be taken by the patient; Identification and organisation of required services and supports; Timeframes and arrangements to review the plan.
Review of a Chronic Disease Management Plan or Coordination of a Review of Team Care Arrangements	732	When reviewing a plan that they are responsible for the GP must: Explain to the patient, and where appropriate the patient’s carer, steps involved in the review; Review all items in the relevant plan with the patient; Make any required amendments to the patient’s plan and ensure they are documented and communicated to the patient; For the Team Care arrangement they must also consult with at least two other health or care providers to review all the matters set out in the relevant plan.

Items used in defining use of Coordinated Care items (Exposure Cohort 2)		

Coordination of a Team Care Arrangement	723	When coordinating a Team Care Arrangement the GP must: Collaborate with at least two other providers who provide different treatment or service types. One can be another medical practitioner; Provide a written plan describing: Treatment and service goals for the patient and how these will be provided by the collaborating parties; Actions to be taken by the patient with adequate explanation of who will be providing the services and how they will be accessed.
Contribution to or review by a general practitioner of a Team Care Arrangement prepared by another provider	729	When reviewing a plan provided by someone else the GP must: Prepare relevant aspects of or make amendments to a multidisciplinary care plan; or Give and record advice provided to another person who is preparing or reviewing a multidisciplinary care plan.
Preparation of a GP Mental Health Treatment Plan for a GP who has not undertaken mental health skills training	2700 (20 minutes), 2701 (40 minutes)	An assessment of the patient must be performed and include: A clinical history (biological, psychological, social) of the problem; A mental state examination; Assessment of co-morbidities and disease risk; Provision of a diagnosis; Where appropriate administer an outcome measurement tool.
Preparation of a GP Mental Health Treatment Plan for a GP who has undertaken mental health skills training	2715 (20 minutes), 2717 (40 minutes)	Plan development must include: Discussing the results of the assessment with the patient; Discussing referral and treatment options including appropriate support services; Developing goals with the patient with agreed actions to be taken by the patient; Where appropriate the provision of psycho-education; Where appropriate the development of a crisis intervention and/or relapse prevention plan Arrangements for appropriate referrals, including treatment, and support services Organisation and documentation of a review date
Review of a GP Mental Health Treatment Plan	2712	The review must include: A review of the patient’s progress towards the goals agreed to in the initial plan; Modifications to the plan if required; Review, reinforcement and expansion of the psych-education; Where appropriate review of the crisis intervention and/or relapse prevention plan or if not previously provided the development of one; Where applicable re-administration of the outcome measurement tool used in the initial assessment.

### Outcomes

The primary outcome is the number of hospital contacts (i.e. hospital admissions plus contacts with the Emergency Department (ED) that do not lead to an admission to prevent double counting of those admitted through the ED) that occur during the outcome period (1.5 – 2.5 years following the index stroke event) (Table 2). Secondary outcomes comprise survival, adherence to medications, and incremental cost per quality adjusted life year (QALY) gained (economic analysis).

### Datasets

#### i. The Australian Stroke Clinical Registry (AuSCR)

The AuSCR is a national clinical quality registry designed to monitor and improve stroke care in Australia . Prospective data are collected on all patients with stroke or TIA admitted to participating hospitals. Cases of stroke in the AuSCR are diagnosed by a clinician, and confirmed with the patient at follow-up. Demographic data, clinical information and receipt of important care types, such as admission to a stroke unit and provision of a comprehensive discharge care plan, are collected. Registrants are followed up at 90-180 days following their admission [15]. At the follow-up interview, registrants are asked about their living situation, and the EQ-5D-3L questionnaire is administered [16]. The EQ-5D-3L is a measure of health status across five dimensions: (1) mobility; (2) self-care; (3) usual activities; (4) pain/discomfort; and (5) anxiety/depression. Respondents are also asked to complete a Visual Analogue Scale (VAS) in which they rate their health related quality of life from 0 (worst imaginable state) to 100 (best imaginable state) [16]. Survival status (date of death and cause of death) is regularly updated through annual linkages with the National Death Index (NDI).

#### ii. The Medicare Benefits Schedule (MBS) claims data

The MBS database contains transactional data and associated dates for all services that are subsidised by the Commonwealth Government under the Medicare Australia scheme [17]. This includes chronic disease management and care coordination Medicare items that can be provided and claimed by a GP to support care planning. Items can also be claimed to allow GPs to update and review existing management or care coordination plans. A large number of other relevant Medicare claim items such as the use of medication management reviews or rehabilitation case conferences are also available. All Medicare claim items have a specific code that can be used to identify the types of care received.

#### iii. The Pharmaceutical Benefits Scheme (PBS)

The PBS database contains a record of medications dispensed that are subsidised by the Australian Commonwealth Government. PBS items can be used to identify whether recommended medications are being used for secondary prevention of stroke (such as antihypertensive, lipid lowering or antithrombotic). Medications such as non-prescribed, privately purchased or those funded under other specialty schemes are not included in the PBS. However, the PBS contains the majority of medications relevant to the study research questions.

#### iv. Admitted patient data (Victoria and Queensland)

State held patient admission datasets include data on all inpatient separations (discharges, transfers and deaths) from all public, private, psychiatric and repatriation hospitals in each state. While the depth of information collected varies between states, all states comply with requirements for the Admitted Patient National Minimum Data Set, enabling cross-jurisdictional comparisons of hospitalisation data [18]. Coding of information by trained clinical coders using standardised International Statistical Classification of Diseases and Related Health Problems, Australian Modification (ICD-AM) and Australian Classification of Health Interventions (ACHI) codes and coding rules also ensures consistency [19].

#### v. Emergency Department (ED) data (Victoria and Queensland)

State held ED datasets include data on presentations to the majority of public EDs. Datasets vary between states in terms of the number and type of variables available as well as the overall reliability and quality of the recorded data, including diagnostic information. Nevertheless a few variables are consistent across datasets and these datasets provide a reliable indication of the number of ED presentations and the primary diagnosis [19].

#### vi. National Aged Care Data Clearing House (NACDC)

The NACDC is a central independent repository of national aged care data and contains data from 1997 onwards. It brings together data related to government-funded aged care programs from a number of sources, including Residential Aged Care; Home Care Packages Programme; Flexible Care; Aged Care Assessment Program; Aged Care Funding Instrument; and the Commonwealth Home Support Programme.

#### vii. Supplementary survey data

A subgroup of AuSCR registrants (N=1,500) will be randomly selected and sent a project specific survey pack at *approximately 2-2.5 years after their initial 3-6 month AuSCR follow-up*. The survey pack includes: (i). the Patient Assessment of Chronic Illness Care+ (PACIC+), a 26 item questionnaire, validated in a chronic disease population, designed to measure specific qualities of care that patients report they have experienced within the healthcare delivery system with a focus on patient centred care [20]; (ii) re-administration of the EQ-5D-3L to enable determining the net change in Quality adjusted life years for the economic evaluation [16]; (iii) the Modified Rankin Scale, a measure of the degree of disability or dependence in the daily activities [21]; (iv) unmet needs using the Long Term Unmet Needs after Stroke (LUNS) screening tool [22] (v) self-reported adherence to secondary prevention medication; and (vi) project specific questions about services accessed and social support. The questions developed for the survey on medication adherence and service access were reviewed by a pharmacist, a general practitioner and a stroke survivor and will be validated against the linked data. These survey data will allow us to obtain a more comprehensive view of patient’s primary care experience and assist in our interpretation of our findings from the administrative data. The survey was reviewed by stroke survivor for readability and relevance.

**Table 2: Datasets and examples of variables likely to be used in the analyses:** *Primary outcome, AuSCR: Australian Stroke Clinical Registry, NDI: National Death Index, GP: General Practitioner; EQ-5D-3L: Euroqol 5 dimension (3 level version)

Datasets	Variables
AuSCR (including NDI linkages)	Cohort identification: age discharge destination place of residence at 3-6 months disability (using EQ-5D-3L) at 90-180 days Outcomes: Date and cause of death long-term quality of life (EQ-5D-3L) (sub-study participants) Covariates: age stroke severity language prior stroke location clinical data socioeconomic strata stroke unit care received a discharge care plan if discharged to home prescribed antihypertensive medication at discharge discharged to in-patient rehabilitation
Medicare Benefits Schedule	Exposures: standard consultations Chronic Disease Management Plans Team Care Arrangements Mental Health Care Plans Covariates: specialists allied health, palliative care pre-stroke usages of chronic disease management, rehabilitation and care coordination primary care items (12 months prior)
Hospital separations	Outcomes: number of admissions* Covariates: comorbidities (previous 5 years) pre-stroke admissions (12 months prior) insurance marital status
Emergency Department	Outcomes: number of contacts* Covariates: *primary diagnosis* *pre-stroke visits (12 months prior)*
Pharmaceutical Benefits Scheme	Outcomes: *medications and dispensing date* Covariates: *comorbidities (based on medication use)*

### Data linkage and merging of datasets

Data from AuSCR registrants will be linked to routinely collected State and Commonwealth data, as well as purposefully collected survey data from a sub cohort of registrants. Permission will be obtained from the relevant data custodians and Human Research Ethics Committees for the study datasets. Implied consent will be obtained from survey participants whereby completion and return of a survey will constitute consent. Participants will also be informed that their survey data will be linked to other health data for the project. Data from Medicare Australia, Pharmaceutical Benefits Scheme (PBS) and National Aged Care Data Clearing House (NACDC) will be linked by staff at the Australian Institute of Health and Welfare (AIHW) data linkage unit.[23] Hospital data will be linked by each of the state data linkage units. Prior data linkage accuracy between AuSCR registrants and these hospital admission datasets was 95% for Victoria using stepwise deterministic methods and 98% for Queensland using probabilistic matching [24].

In Australia, unique identifiers are unavailable. A two stage separation model of data linkage will be used, in which AuSCR identifying data in the form of *full name, full residential address, date of birth, sex and date of death (if applicable)* for the study cohort will be submitted to participating data linkage units along with a project specific person identifier [25]. The de-identified content data will be submitted by the data custodians into the Sax Institute’s Secure Unified Research Environment (SURE), for merging using a pre-specified project ID for each AuSCR registrant. SURE is a remote-access computing environment that must be used to access data that are linked and held by the AIHW. All data are stored on SURE-dedicated servers in the SURE facility and located in locked cabinets, and housed in a data centre, within the Sax Institute, called ‘Global Switch’. Global Switch is located in Sydney and is listed on the Australian Government Data Centre Facilities Panel. Data are accessed through a virtual project workspace and restricted to investigators listed on the Ethics Committee application. De-identified project specific surveys will also be submitted into SURE for merging with the other datasets using the Project ID. All analyses will be undertaken within this secure system by approved researchers.

### Data analysis

#### (1) Cohort stratification

The primary analyses will be performed using data from registrants who completed the EQ-5D-3L questionnaire at the AuSCR 90-180 day follow-up. They will be stratified into two groups based on their responses: *(1) without disability; and (2) with disability*. An algorithm developed by the investigators will be used to identify those with ongoing disability severe enough to impact on their quality of life [26]. This is defined as having a problem in one or more of the five EQ-5D-3L domains and a clinically important (eight point or more) lower VAS score than what would be expected for people in the general population of a similar age [16, 27].

#### (2) Exposure periods

The index event will be the first stroke episode recorded in the AuSCR. Time zero (commencement of follow-up) will be 6 months following participants’ index stroke admission date (Figure 1). By this time, it is expected that patients will have completed their rehabilitation and will be primarily under the care of their general practitioner (GP). It is also likely that information obtained from the AuSCR 3-6 month follow-up interview will be available for stratification. The first 12 months after time zero will be used to define the exposure groups outlined in Figure 1 and the next 12 months (i.e. 1.5-2.5 years after stroke) will be used to measure the primary outcomes.

#### (3) Exposure groups

There are two primary exposures of interest: (i) chronic disease management and (2) care coordination which includes mental health management (Table 1). Exposure group one will be defined as: (i) having one or more Medicare claim item for an initiation or review of a Chronic Disease Management Plan during the exposure period as defined above; and (ii) GP coordinated multidisciplinary care as indicated by having one or more Medicare claim item for initiation or review of a Team Care Arrangement or Mental Health Care Plan (Figure 2).

**Figure 3: Flow chart of stratified study design:**
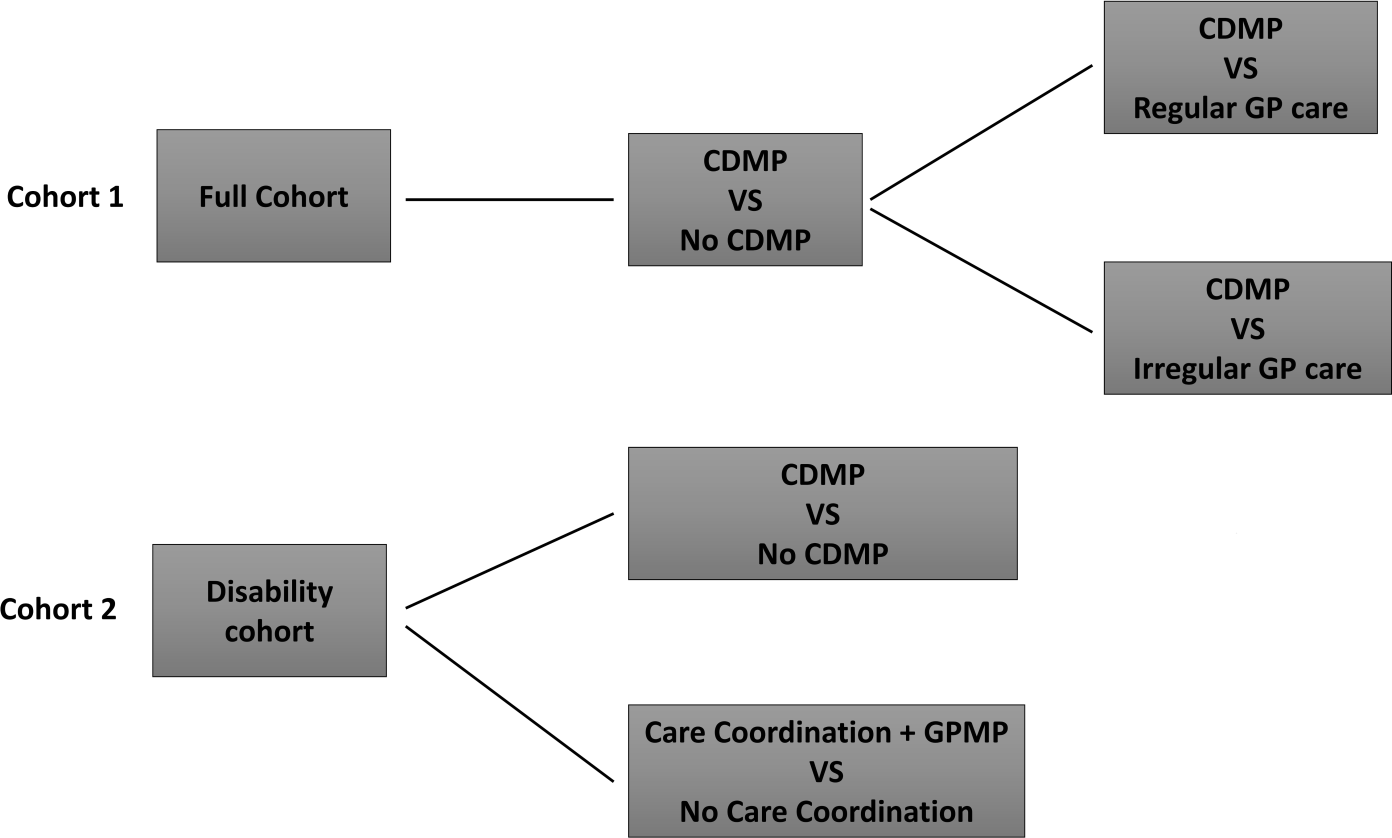
GP: General Practitioner; CDMP: Chronic disease management plan

The cohort for each of the exposure groups will vary to account for appropriateness of care. A Chronic Disease Management Plan or review (cohort one) will be considered an optimal pathway for all participants for addressing secondary prevention needs through a structured management approach. A Team Care Arrangement or review (cohort 2) may not be suitable for all participants, but may facilitate optimal management for those with complex healthcare needs who require multidisciplinary care as recommended in the Australian Stroke Clinical Guidelines (14), such as those with ongoing disability identified through the EQ-5D-3L responses.

#### (4) Control groups

The primary control group will be standard GP care that does not involve a Medicare claim for a chronic disease management or care coordination item. A sub analysis will also be performed for each of the exposure groups whereby the control group will be restricted to those who received regular GP care.

#### (5) Other definitions

GP regularity will be defined using a validated regularity index based on the variance of the distribution of days between visits [28]. Scores will be dichotomised using a majority of care definition with a score of ≥75% defined as regular [29]. Sensitivity analyses will be performed for other quartile cut points, by different intervals, e.g. monthly or quarterly contacts and using a variance index to adjust for the potential confounding effect of frequency on variance [30].

Hospital contacts will be defined using a 24-hour rule in which separations that occur within a 24-hour period (e.g. due to moving between hospitals, wards or from the ED to an admission) are combined into a single event to prevent over counting.

We will us the landmark analysis method to estimate the association between secondary prevention medication adherence and outcomes such as mortality and hospital contacts [31].Recommended medication dosages per day will be determined based on consumer’s medicines information (available from www.mimsonline.com.au/) and in consultation with an interdisciplinary working group comprising clinicians, pharmacists and researchers. Adherence will be calculated using the proportion of days covered (PDC) from first medication supply date after discharge from hospital to six-months and one-year after discharge (two landmark dates).

The numerator will be the number of days a patient had access to medication from the first supply date until the landmark dates including all supplies. The denominator will be the number of days from first supply date to the landmark dates [32, 33]. Users for each drug group will be defined as having at least two supplies for that medication (see equation to calculate PDC Figure 4).

Persistence in use of medication will be defined as “the duration of time from initiation to discontinuation of therapy” [34, 35]. Discontinuation will be defined as the absence of any dispensing for a period of at least three times the number of pills last dispensed (assuming a dose of one pill/day) plus a 5-day grace period.

**Figure 4: Formula used to calculate medication adherence:** $\mathrm{PDC}=\frac{d_{\mathrm{supplied}}}{d_{\mathrm{period}}}\times 100\%$

*d_supplied_* = number of days with ≥ 1 unit of medication available between *t_0_* and *t_landmark_*

*d_period_* = number of days between *t_0_* and *t_landmark_*

*t_landmark_* = six months and one year after date of discharge from hospital

*t_0_* = date of first medication supply after *t_landmark_*

Comorbidities will be identified using International Classification of Diseases 10^th^ revision (ICD-10) codes obtained from the hospital dataset. Diagnostic coding (up to 42 diagnoses per admission) for all admissions in the 5-years prior to, and including, the index stroke event will be used for detection of chronic disease co-morbidities based on any recording of a related ICD-10 code (any level) during this period [36, 37]. Comorbidity codes will be identified and coded based on the Elixhauser Index, a comorbidity index with >30 items commonly used in research on stroke and other chronic diseases [38].

#### (6) Primary outcome analysis – aim 1

Differences in rates (person-time denominator) for hospital presentations per year (combined ED and hospital presentations) between exposure groups will be determined for the period 1-2 years after time zero (i.e. 1.5-2.5 years post stroke onset) and censored for deaths. As there may be valid clinical reasons for prescribing different types of care for different patients, propensity score matching will be used to minimise confounding by indication for known confounders [39, 40]. A propensity score, based on demographic and clinical characteristics, will be calculated for each patient to estimate the probability that they will receive a certain care type. Propensity scores will be categorised into quintiles. Each quintile will be assessed for balance in characteristics of patients in the different care pathways and quintiles with imbalance removed. This method will allow us to maximise the patient sample and the external validity of the findings [41].

The primary outcome of hospital contacts involves count data and so, based on the dispersion of the data, either multivariable Poisson or negative binomial regression for multiple events will be used. The model will be adjusted by the propensity score and used to test for differences in the rates of hospital presentations between exposure groups (Figure 3). Clustering by State, Local Government Area (LGA) and the hospital at which patients had their admission for AuSCR will be assessed and, if present, we will use multilevel analyses.

Sensitivity analyses will be applied to help us better understand and assess the possibility of misclassification of the exposure, selection bias, variability in costs, and unmeasured confounding [42]. Descriptive analyses will be applied to data from the survey and NACDC to help us better understand alternate care pathways and the context within which the different models of primary care may, or may not, have been used. These analyses will also allow us to better understand those who are most likely to benefit from the different types of care.

#### (7) Secondary outcome analysis

##### Survival

Cox proportional hazards regression will be used to assess differences between groups for time to death.

##### Medication adherence and persistence – aim 2

The landmark analysis method described above will be used to estimate association between PDC and outcomes [31]. The influence of persistence and adherence on long-term outcomes (hospital contacts and survival) independent of the primary care types received will also be assessed using methods similar to those described above. As stated in the Australian Stroke Clinical Guidelines all patients should be prescribed antihypertensive and lipid lowering medications and all except those with an ICH should receive antithrombotic medications unless contra-indicated [6]. Relevant comorbidities will be considered when interpreting the results.

##### Economic analysis - aim 3

A patient-level (micro-simulation) decision analytic model that will include various input values derived from the patent-level data and other sources of cost information will be developed based on a government perspective as purchaser and provider of healthcare and a time horizon of 2.5 years post-stroke [43]. We will discount costs and outcomes that occur after 12 months by 5% [44]. Costs will be adjusted, i.e. inflated, to a common reference year (2016-2017).

Costs of community-based care attributable to the stroke will be estimated by comparing the healthcare utilisation in the two years prior to the stroke and the healthcare utilisation between 0.5 to 2.5 years following the stroke. This time period has been chosen in order to exclude the costs of stroke prior to exposure to the different chronic disease management and care coordination interventions. Costs of subsequent hospital readmissions and ED visits will be calculated for each person in the cohort by applying unit prices extracted from the best available Australian sources. This will include direct, indirect (where available) and total costs for services such as allied health, theatre costs, intensive care and imaging. Costs of medications prescribed and Medicare claims will be calculated by applying unit prices on individual claim items published in the main reference sources (e.g. MBS or PBS). An expert reference group will be consulted when we need to make assumptions about cost values or resource estimates.

For the different exposure groups that will be compared, the net change in quality of life will be estimated from the responses to the EQ-5D-3L questionnaire between the AuSCR follow-up (90 to 180 days after stroke) and the time of the supplementary survey for this study (conducted at approximately 2-2.5 years after stroke). QALYs will then be estimated by applying Australian-derived utilities to the responses to the EQ-5D-3L questionnaire to estimate the years lived with disability (YLD) and from calculations of years of life lost (YLL) based on participants’ age at death and the Australian life expectancy tables for different age groups published by the Australian Bureau of Statistics. Incremental costs and benefits (QALYs gained, hospital presentations avoided) of the various pathways of care by exposure group will be determined from the economic model.

The use of the different models of primary care will be deemed cost-effective compared to standard GP care if the incremental cost per QALY gained is less than the national annual gross domestic product per capita (approximately $50,000 in Australia) [45]. We will also calculate the incremental cost per (i) hospital admission avoided and (ii) best practice use of stroke prevention medication with the use of chronic disease management and care coordination primary care items compared to standard models of care. Sensitivity and probabilistic multivariable uncertainty analyses will be performed to assess the robustness of results using plausible ranges for the parameters that most strongly influence the costs or outcome.

#### (8) Sample size

Likely rates of hospital contacts per year were calculated using data a from a prior linkage study [46]. Publically available Medicare data from 2016 were used to estimate uptake of Chronic Disease Management Plans (approximately 50%) and Team Care Arrangements or Mental Health Care Plans (approximately 33%) [47]. Our predicted sample size of over 25,000 (full cohort, primary outcome; α>0.05) will allow us to detect a 6-8% difference in the number of hospital contacts between those who did and did not receive a Chronic Disease Management Plan and those with disability who did and did not receive a Team Care Arrangement or Mental Health Care Plan.

### Interpretation and dissemination of results

We will establish a multidisciplinary reference committee of national experts in general practice, stroke, chronic disease, aged care, allied health as well as government representatives and stroke survivors and their caregivers. Members will be recruited through the multidisciplinary investigator team and their organisational networks. This committee will provide a systematic interpretation of the results focused on implications for policy decision-making. Discussions about the likely types of unmeasured confounders and how these may have influenced the results will also take place. The comparative effectiveness and cost-effectiveness results will be reviewed and contrasted against a range of implementation and other policy considerations. The committee will finalise, initially through open discussion, a range of issues or criteria they consider relevant to policy decision-making and implementation over and above the study results. This could include, but is not limited to, issues of ‘acceptability to stakeholders’, ‘strength of the evidence’, ‘impact on health inequalities’ and ‘feasibility of implementation’. Depending on the outcomes of these deliberations, the committee members may then be involved in a formal elicitation process to weight their preferences to permit quantitative ranking of the recommendations using multi-criteria decision analysis methods [48, 49].

## Conclusion

This study will provide a large scale evaluation of the effectiveness and cost-effectiveness of Medicare funded policies for chronic disease management and care coordination primary care items in managing patients with stroke. Recommendations from the reference committee will help shape how these items are used in the future. These will be applicable to most people with multi-morbidity and/or chronic disability conditions given that stroke is a major contributor to both disability and morbidity. If these primary care items are shown to be effective, our findings will have a great potential to modify uptake and facilitate expansion of these programs by government. The methods, analyses and breadth of datasets employed for this project will help advance the use of data linkage for healthcare evaluation [50].

## Abbreviations

ACHI	Australian Classification of Health Interventions
AIHW	Australian Institute of Health and Welfare
AuSCR	Australian Stroke Clinical Registry
DALY	Disability Adjusted Life Year
DDD	Defined Daily Dose
ED	Emergency Department
EQ-5D-3L	Euroqol 5 dimension questionniare (3 level version)
GP	General Practitioner
ICD-AM	International Statistical Classification of Diseases and Related Health Problems, Australian Modification
LGA	Local Government Area
LUNS	Long Term Unmet Needs after Stroke
MBS	Medicare Benefits Schedule
NACDC	National Aged Care Data Clearing House
NDI	National Death Index
PACIC+	Patient Assessment of Chronic Illness Care Plus
PBS	Pharmaceutical Benefits Scheme
PDC	Proportion of Days Covered
PRECISE	Evaluation of Enhanced Models of Primary Care in the Management of Stroke and Other Chronic Disease
QALY	Quality Adjusted Life Year
RECORD	REporting of studies Conducted using Observational Routinely-collected health Data
SURE	Secure Unified Research Environment
VAS	Visual Analogue Scale

## References

[1] Feigin VL, Norrving B, Mensah GA. Global Burden of Stroke. Circ Res. 2017;120(3):439-48. 10.1161/CIRCRESAHA.116.308413 28154096

[2] Thrift AG, Howard G, Cadilhac DA, et al. Global stroke statistics: An update of mortality data from countries using a broad code of “cerebrovascular diseases”. Int J Stroke. 2017;12(8):796-801. 10.1177/1747493017730782 28895807

[3] Mohan KM, Wolfe CD, Rudd AG, et al. Risk and cumulative risk of stroke recurrence: a systematic review and meta-analysis. Stroke. 2011;42(5):1489-94. 10.1161/STROKEAHA.110.602615 21454819

[4] Andrew N, Kilkenny M, Naylor R, et al. Understanding long-term unmet needs in Australian survivors of stroke. Int J Stroke. 2014;9 (Suppl A100:):106-12. 10.1111/ijs.12325 25042019

[5] Gloede TD, Halbach SM, Thrift AG, et al. Long-term costs of stroke using 10-year longitudinal data from the North East Melbourne Stroke Incidence Study. Stroke. 2014;45(11):3389-94. 10.1161/STROKEAHA.114.006200 25342028

[6] Guidelines Content Working Party Stroke Foundation. Clinical Guidelines for Stroke Management 2017. Melbourne: Stroke Foundation; 9 2018.

[7] Thrift AG, Kim J, Douzmanian V, et al. Discharge is a critical time to influence 10-year use of secondary prevention therapies for stroke. Stroke. 2014;45(2):539-44. 10.1161/STROKEAHA.113.003368 24335222

[8] Gillam S. Financial incentive schemes in primary care. J Healthc Leadersh. 2015;7:75-80. 10.2147/JHL.S64365 29355191 PMC5740997

[9] Minchin M, Roland M, Richardson J, et al. Quality of Care in the United Kingdom after Removal of Financial Incentives. N Engl J Med 2018;379(10):948-57. 10.1056/NEJMsa1801495 30184445

[10] Adaji A, Schattner P, Jones KM, et al. Care planning and adherence to diabetes process guidelines: medicare data analysis. Aust Health Rev. 2013;37(1):83-7. 10.1071/AH11136 23157923

[11] Vitry AI, Nguyen TA, Ramsay EN, et al. General practitioner management plans delaying time to next potentially preventable hospitalisation for patients with heart failure. Intern Med J. 2014;44(11):1117-23. 10.1111/imj.12512 24942781

[12] Benchimol EI, Smeeth L, Guttmann A, et al. The REporting of studies Conducted using Observational Routinely-collected health Data (RECORD) statement. PLoS Med. 2015;12(10):e1001885. 10.1371/journal.pmed.1001885 26440803 PMC4595218

[13] Australian Government Department of Health. MBS online - Medicare Benefits Schedule Canberra, Australia: Commonwealth of Australia; 2018 [2018]. http://www9.health.gov.au/mbs/search.cfm].

[14] Cant RP, Foster MM. Investing in big ideas: utilisation and cost of Medicare Allied Health services in Australia under the Chronic Disease Management initiative in primary care. Aust Health Rev. 2011;35(4):468-74. 10.1071/AH10938 22126951

[15] Cadilhac DA, Lannin NA, Anderson CS, et al. Protocol and pilot data for establishing the Australian Stroke Clinical Registry. Int J Stroke. 2010;5(3):217-26. 10.1111/j.1747-4949.2010.00430.x 20536618

[16] The EuroQol Group’s International Task Force on Self-Reported Health. Measuring Self-Reported Population Health: An International Perspective based on EQ-5D. Rotterdam: EuroQol Research Foundation, 2004.

[17] Australian Government Department of Health. Medicare Benefits Schedule Book. Canberra (Aust): Commonwealth of Australia, 2017.

[18] Australian Institute of Health and Welfare. Admitted patient care national minimum data sets 2016-17 2017. https://meteor.aihw.gov.au/content/index.phtml/itemId/612171].

[19] Andrew NE, Sundararajan V, Thrift AG, et al. Addressing the challenges of cross-jurisdictional data linkage between a national clinical quality registry and government-held health data. Aust N Z J Public Health. 2016;40(5):436-42. 10.1111/1753-6405.12576 27625174

[20] Glasgow RE, Wagner EH, Schaefer J, et al. Development and validation of the Patient Assessment of Chronic Illness Care (PACIC). Med Care. 2005;43(5):436-44 10.1097/01.mlr.0000160375.47920.8c 15838407

[21] Lahiri S, Kamel H, Meyers EE, et al. Patient-Powered Reporting of Modified Rankin Scale Outcomes Via the Internet. Neurohospitalist. 2016;6(1):11-3. 10.1177/1941874415593760 26753052 PMC4680902

[22] LoTS care Luns study team. Validation of the longer-term unmet needs after stroke (LUNS) monitoring tool: a multicentre study. Clin Rehabil. 2013;27(11):1020-8. 10.1177/0269215513487082 23787941

[23] Boyd J, Ferrante A, O’Keefe C, et al. Data linkage infrastructure for cross-jurisdictional health-related research in Australia. BMC Health Serv Res. 2012;12:480. 10.1186/1472-6963-12-480 23272652 PMC3579698

[24] Kilkenny M, Kim J, Andrew N, et al. Maximising use of data and avoiding data waste: case study from stroke. Med J Aust. 2019;210:27-31. 10.5694/mja2.12029 30636305

[25] Kelman C, Bass A, Holman C. Research use of linked health data - a best practice protocol. Aus NZ J Public Health. 2002;26:251-5 10.1111/j.1467-842X.2002.tb00682.x 12141621

[26] Andrew N, Kilkenny M, Lannin N, et al. Is health related quality of life between 90 and 180 days following stroke associated with long-term unmet needs? Qual Life Res. 2016;25:2053-62. 10.1007/s11136-016-1234-5 26847339

[27] Kim SK, Kim SH, Jo MW, et al. Estimation of minimally important differences in the EQ-5D and SF-6D indices and their utility in stroke. Health Qual Life Outcomes. 2015;13:32. 10.1186/s12955-015-0227-3 25889191 PMC4359514

[28] Gibson DA, Moorin RE, Preen D, et al. Enhanced primary care improves GP service regularity in older patients without impacting on service frequency. Aust J Prim Health. 2012;18(4):295-303. 10.1071/PY11050 22951191

[29] Menec VH, Sirski M, Attawar D, et al. Does continuity of care with a family physician reduce hospitalizations among older adults? J Health Serv Res Policy. 2006;11(4):196-201. 10.1258/135581906778476562 17018192

[30] Youens D, Moorin R, Preen D, et al. Regular General Practitioner contact - analysis of methods for measurementusing administrative data. IJPDS. 2018;3:267 10.23889/ijpds.v3i4.858

[31] Dafni U. Landmark analysis at the 25-year landmark point. Circ Cardiovasc Qual Outcomes. 2011;4(3):363-71. 10.1161/CIRCOUTCOMES.110.957951 21586725

[32] Martin BC, Wiley-Exley EK, Richards S, et al. Contrasting measures of adherence with simple drug use, medication switching, and therapeutic duplication. Ann Pharmacother. 2009;43(1):36-44. 10.1345/aph.1K671 19126828

[33] Raebel MA, Schmittdiel J, Karter AJ, et al. Standardizing terminology and definitions of medication adherence and persistence in research employing electronic databases. Med Care. 2013;51(8 Suppl 3):S11-21. 10.1097/MLR.0b013e31829b1d2a 23774515 PMC3727405

[34] Schaffer A, Buckley N, Dobbins T, et al. The crux of the matter: Did the ABC’s Catalyst program change statin use in Australia? Med J Aust. 2015;202:591-5. 10.5694/mja15.00103 26068693

[35] Cramer JA, Roy A, Burrell A, et al. Medication compliance and persistence: terminology and definitions. Value Health. 2008;11(1):44-7. 10.1111/j.1524-4733.2007.00213.x 18237359

[36] Gattellari M, Goumas C, Aitken R, et al. Outcomes for patients with ischaemic stroke and atrial fibrillation: the PRISM study (A Program of Research Informing Stroke Management). Cerebrovasc Dis. 2011;32(4):370-82. 10.1159/000330637 21921601

[37] Preen DB, Holman CD, Spilsbury K, et al. Length of comorbidity lookback period affected regression model performance of administrative health data. J Clin Epidemiol. 2006;59(9):940-6. 10.1016/j.jclinepi.2005.12.013 16895817

[38] Quan H, Sundararajan V, Halfon P, et al. Coding algorithms for defining comorbidities in ICD-9-CM and ICD-10 administrative data. Med Care. 2005;43(11):1130-9 10.1097/01.mlr.0000182534.19832.83 16224307

[39] Austin PC. An Introduction to Propensity Score Methods for Reducing the Effects of Confounding in Observational Studies. Multivariate Behav Res. 2011;46(3):399-424. 10.1080/00273171.2011.568786 21818162 PMC3144483

[40] Rosenbaum P, Rubin D. Reducing Bias in Observational Studies Using Subclassification on the Propensity Score. J Am Stat Assoc. 1984;79:516-24. 10.2307/2288398

[41] Andrew N, Kim J, Thrift A, et al. Prescription of antihypertensive medication at discharge influences survival following stroke. Neurology. 2018;90:1-9. 10.1212/WNL.0000000000005023 29386279

[42] Orsini N, Bellocco R, Bottai M, et al. A tool for deterministic and probabilistic sensitivity analysis of epidemiologic studies. Stata Journal. 2008;8:29-48 10.1177/1536867X0800800103

[43] Mitra N, Indurkhya A. A propensity score approach to estimating the cost-effectiveness of medical therapies from observational data. Health Econ. 2005;14(8):805-15. 10.1002/hec.987 15791679

[44] Australian Government Department of Health. Pharmaceutical Benefits Advisory Committee Guidelines 2018. https://pbac.pbs.gov.au/section-3a/a-1-overview-and-rationale-of-economic-evaluation.html].

[45] Marseille E, Larson B, Kazi DS, et al. Thresholds for the cost–effectiveness of interventions: alternative approaches. Bull World Health Organ. 2015;93(2):118-24. 10.2471/blt.14.138206 25883405 PMC4339959

[46] Cadilhac DA, Andrew NE, Kilkenny MF, et al. Improving quality and outcomes of stroke care in hospitals: Protocol and statistical analysis plan for the Stroke123 implementation study. Int J Stroke. 2017;13:96-106. 10.1177/1747493017730741 28914187

[47] Australian Government Department of Human Services. Medicare Item Reports: Medicare Australia Statistics; 2016 [cited 2017]. http://medicarestatistics.humanservices.gov.au/statistics/mbs_item.jsp].

[48] Churilov L, Liu D, Ma H, et al. Multiattribute selection of acute stroke imaging software platform for Extending the Time for Thrombolysis in Emergency Neurological Deficits (EXTEND) clinical trial. Int J Stroke. 2013;8(3):204-10. 10.1111/j.1747-4949.2012.00787.x 22812698

[49] Craig LE, Churilov L, Olenko L, et al. Testing a systematic approach to identify and prioritise barriers to successful implementation of a complex healthcare intervention. BMC Med Res Methodol. 2017;17(1):24. 10.1186/s12874-017-0298-4 28173749 PMC5297164

[50] McGrail K, Jones K, Akbari A, et al. A Position Statement on Population Data Science: The Science of Data about People. IJPDS. 2018;3:4. 10.23889/ijpds.v3i1.415 PMC8142960 34095517

